# Chemoinformatic Analysis of Selected Cacalolides from *Psacalium decompositum* (A. Gray) H. Rob. & Brettell and *Psacalium peltatum* (Kunth) Cass. and Their Effects on FcεRI-Dependent Degranulation in Mast Cells

**DOI:** 10.3390/molecules23123367

**Published:** 2018-12-19

**Authors:** Jorge Iván Castillo-Arellano, Juan Carlos Gómez-Verjan, Nadia A. Rojano-Vilchis, Myrna Mendoza-Cruz, Manuel Jiménez-Estrada, Héctor E. López-Valdés, Hilda Martínez-Coria, Roger Gutiérrez-Juárez, Claudia González-Espinosa, Ricardo Reyes-Chilpa, Isabel Arrieta-Cruz

**Affiliations:** 1 Pharmacobiology Department, Center for Research and Advanced Studies of the National Polytechnic Institute, Mexico City 14330, Mexico; jorge.ivan@ciencias.unam.mx; 2 Department of Natural Products, Institute of Chemistry, National Autonomous University of Mexico, Mexico City 04510, Mexico; nadiarojano@yahoo.com.mx (N.A.R.-V.); manuelj@unam.mx (M.J.-E.); 3 Department of Basic Research, National Institute of Geriatrics, Ministry of Health, Mexico City 10200, Mexico; carlosverjan132@gmail.com; 4 Botanical Garden, Institute of Biology, National Autonomous University of Mexico, Mexico City 04510, Mexico; myrna@ib.unam.mx; 5 Division of Research, Faculty of Medicine, National Autonomous University of Mexico, Mexico City 04510, Mexico; helopezv@gmail.com (H.E.L.-V.); hildamcoria@gmail.com (H.M.-C.); 6 Department of Biomedical Sciences, Faculty of High Studies Zaragoza, National Autonomous University of Mexico, Mexico City 09230, Mexico; roger.gutierrez@zaragoza.unam.mx

**Keywords:** *Psacalium decompositum*, *Psacalium peltatum*, cacalol, calcium channels, maturin, reactive oxygen species, inflammation

## Abstract

Cacalolides are a kind of sesquiterpenoids natural compounds synthesized by *Psacalium decompositum* (A. Gray) H. Rob. & Brettell or *Psacalium peltatum* (Kunth) Cass. Antioxidant and hypoglycemic effects have been found for cacalolides such as cacalol, cacalone or maturine, however, their effects on inflammatory processes are still largely unclear. The main aim of this study was to investigate the biological activities of secondary metabolites from *P. decompositum* and *P. peltatum* through two approaches: (1) chemoinformatic and toxicoinformatic analysis based on ethnopharmacologic background; and (2) the evaluation of their potential anti-inflammatory/anti-allergic effects in bone marrow-derived mast cells by IgE/antigen complexes. The bioinformatics properties of the compounds: cacalol; cacalone; cacalol acetate and maturin acetate were evaluated through Osiris DataWarrior software and Molinspiration and PROTOX server. In vitro studies were performed to test the ability of these four compounds to inhibit antigen-dependent degranulation and intracellular calcium mobilization, as well as the production of reactive oxygen species in bone marrow-derived mast cells. Our findings showed that cacalol displayed better bioinformatics properties, also exhibited a potent inhibitory activity on IgE/antigen-dependent degranulation and significantly reduced the intracellular calcium mobilization on mast cells. These data suggested that cacalol could reduce the negative effects of the mast cell-dependent inflammatory process.

## 1. Introduction

Mast cells are key effectors of type I hypersensitivity reactions (allergies) and other inflammatory reactions, since they secrete numerous pre-formed and *de novo* synthesized pro-inflammatory mediators that promote endothelial activation, smooth muscle contraction and chemotaxis of other immune cells to the site where mast cells are activated [1]. Allergic inflammation differs from other innate inflammatory reactions in that the first are intense, rapid, and can lead to complex life-threatening conditions such as asthma and anaphylaxis [2]. The main mechanism of mast cells activation in allergy is the crosslinking of the high affinity immunoglobulin E (IgE) receptor (FcεRI) with IgE/antigen (IgE/Ag) complexes. FcεRI signal transduction requires the production of reactive oxygen species (ROS) and calcium mobilization, together with the activation of selected kinases to trigger degranulation and the consequent release of histamine, β-hexosaminidase, serotonin and tumor necrosis factor (TNF), in addition to the *de novo* production of prostaglandins, leukotrienes and numerous cytokines [3]. IgE/Ag-dependent degranulation and cytokine synthesis of mast cells have been recognized as relevant pharmacological targets for the control of allergic reactions [4]. However, despite the importance of allergic diseases worldwide, appropriate pharmacological control of mast cells degranulation has not been achieved yet [5]. The FcεRI-dependent degranulation in mast cells is an appropriate model to study the effects of new natural products on inflammation. In fact, some natural compounds have been tested in models of mast cells-dependent inflammation; for example, we have recently described that the xanthone jacareubin, a natural product from the tropical tree *Calophyllum brasiliense* displays potent anti-allergic and anti-inflammatory activities in degranulation of mouse mast cells [6].

On the other hand, *Psacalium decompositum* (A. Gray) H. Rob. & Brettell, belonging from Asteraceae family is a wild herb from the pine forests of Northwest Mexico, commonly known as “matarique” and used locally as a remedy in folk medicine. The Raramuri people and peasants of the State of Chihuahua, use a decoction of the roots and rhizome of this species for the treatment of rheumatic disorders, pain, hepatic and renal colic, neuralgia, ulcers and colds. The roots and rhizome contain a number of sesquiterpenoids cacalolides such as cacalol, cacalone, epicacalone, maturine, 3-hydroxycacalolide, epi-3-hydroxycacalolide [7,8,9]. Additionally, *P. decompositum* is currently used by the urban populations of Mexico as an antidiabetic remedy; in fact, several studies have examined the hypoglycemic properties of cacalol and other cacalolides [8]. Interestingly, the decoction of the roots from *P. decompositum* has shown clear hypoglycemic effects in healthy mice and in transtiently hyperglycemic rabbits [7]. Besides, polysaccharide fractions obtained from the freeze-dried water extract from *P. decompositum* significantly reduced fasting blood glucose in mild alloxan-diabetic mice suggesting that the aqueous fraction, containing fructan-type oligosaccharides, is responsible for the hypoglycemic effects observed [8,10]. Recently, treatment with a fructooligosaccharides fraction from *P. decompositum* was shown to significantly reduce cholesterol, triglycerides, IL-6, IFN-γ, MCP-1, IL-1β and VEGF levels, as well as a decrease in body weight in an animal model of obesity. These observations suggest novel anti-inflammatory and hypolipidemic properties of the fructooligosaccharides fraction [11]. Meanwhile, the hexane extract and two compounds, cacalol and cacalone isolated from *P. decompositum*, have been shown to inhibit inflammation in the carrageenan-induced edema of the rat paw; these compounds also exhibited a dose-dependent anti-inflammatory activity in the TPA-induced mouse ear edema. In these models cacalone had the most prominent activity [12]. 

In medicinal plant markets of Mexico City, *Psacalium peltatum* (Kunth) Cass., is a common substitute for *P. decompositum*, because the former grows in the nearby pine forests. *P. peltatum* is also kown as “matarique” and the roots and rhizome also contain cacalolides, such as maturin, maturin acetate, and maturinin, but not cacalol or cacalone. Few studies have shown the potential effects of maturine acetate in the inflammatory process; maturine acetate reduces the production of pro-inflammatory cytokines (TNF-α and IL-1β) by lypopolysaccharide (LPS)-activated peritoneal macrophages. Maturine acetate also stimulates the proliferation of murine macrophages and splenocytes, induces lysosomal enzyme activity, pinocytosis and NK cell activity, as well as increases the release of IL-2, IL-15 and IFN-γ in immunosuppressed mice, showing relevant immunostimulatory activities [13,14]. 

Despite all the available literature, there is currently no information about the physicochemical, pharmacokinetic, medicinal chemistry and toxicoinformatic properties of some cacalolides. Thus, we aimed to examine the biological activities of secondary metabolites from *P. decompositum* and *P. peltatum* through two not mutually exclusive approaches: (1) bioinformatic analysis: chemoinformatic and toxicoinformatic; and (2) pharmacological investigations based on ethnomedical use. 

Chemoinformatic and toxicoinformatic have demonstrated to be reasonable alternatives for the early estimation of absorption, distribution, metabolism, excretion and toxicity (ADMET) properties and represent substantial time and cost reductions during the drug discovery phase [15]. This is the reason why in recent years several groups have used this type of computational methodologies to improve and accelerate drug design. 

With the aim of providing detailed information about of the compounds, in this work we performed a chemoinformatic and toxicoinformatic analysis of four cacalolides: cacalol, cacalol acetate, cacalone and maturin acetate isolated from *P. decompositum* and *P. peltatum.* Besides, we described a potential anti-inflammatory/anti-allergic activity of these compounds on mast cells not previously reported. Furthermore, we postulate a possible molecular mechanism for the inhibition of inflammation by these compounds.

## 2. Results

### 2.1. Chemoinformatic and Toxicoinformatic Analysis of Cacalolides from P. decompositum and P. peltatum

**Figure 1:**
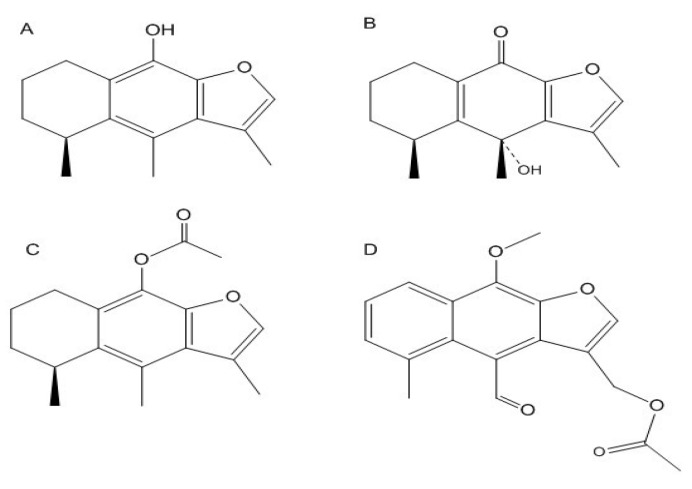
Chemical structures of cacalolides from two species of matarique (*P. decompositum* and *P. peltatum*): (**A**) cacalol; (**B**) cacalone; (**C**) cacalol acetate; (**D**) maturin acetate.

The bioinformatic properties of the cacalolides: cacalol, cacalone, cacalol acetate and maturin acetate (Figure 1) were examined. Among all cacalolides only cacalol displayed high water solubility conferring it some advantages such as a good drug gastrointestinal absorption and adequate blood-brain barrier penetration. According to Lipinski, Ghose and Lead-likenes classification, cacalol had the best score regarding bioavailability, biosynthesis and pharmacokinetics. Interestingly, all studied cacalolides exhibited a very low toxicity and in no case mutagenic or tumorigenic effects were detected (Table 1). 

Because cacalol showed interesting physicochemical, pharmacokinetic and toxicoinformatic properties among all analyzed cacalolides, we next attempted to indentify its most important biological targets through the use of an in silico approach. We found that some molecules involved in the regulation of the immune system could potentially interact with cacalol including: Fc epsilon receptor, MAPK signaling, PI3K-AKT pathway and nerve growth factor (Table 2).

### 2.2. Experimental Validation of Anti-Inflammatory/Anti-Allergic Activity

To validate the anti-inflammatory/anti-allergic properties of cacalol that were predicted by bioinformatic analysis, we performed in vitro pharmacological assays using a very well established IgE/Antigen-dependent degranulation cellular model in bone marrow-derived mast cells (BMMCs).

#### 2.2.1. Cacalolides Inhibit Mast Cell Degranulation Activated by FcεRI Triggering In Vitro

In order to validate the potential inhibitory activity of tested cacalolides, their effect on FcεRI-dependent activation of BMMCs was evaluated, taking advantage of this cellular model that has been widely utilized to determine the potential anti-inflammatory activity of a number of natural and synthetic compounds [4,5].

**Figure 2:**
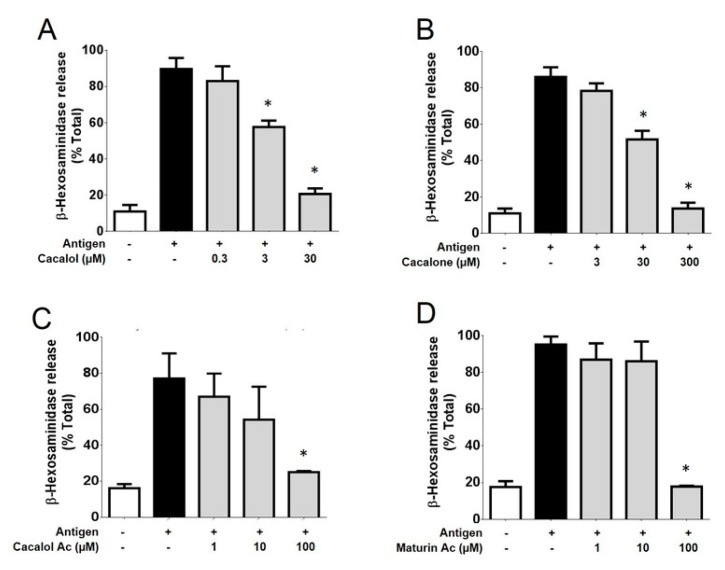
Cacalolides inhibit degranulation stimulated by antigen in bone marrow-derived mast cells. Effect of cacalol (**A**) cacalone (**B**) cacalol acetate (**C**) and maturine acetate (**D**) pre-treatment on DNP-HSA-induced degranulation, *n* = 3. * *p* ≤ 0.05.

Among the four compounds evaluated, cacalol showed the highest inhibitory activity on IgE/Antigen-dependent degranulation. Cacalol, at 30 µM, produced an 88% inhibition of degranulation, followed by maturine acetate and cacalol acetate which, at 100 µM concentrarion, caused an inhibition of almost 100% and 85%, respectively. Finally, cacalone produced a blockage close to 100% at a concentration of 300 µM (Figure 2). 

#### 2.2.2. Cacalol Interfered with FcεRI-Induced Intracellular Calcium Mobilization

**Figure 3:**
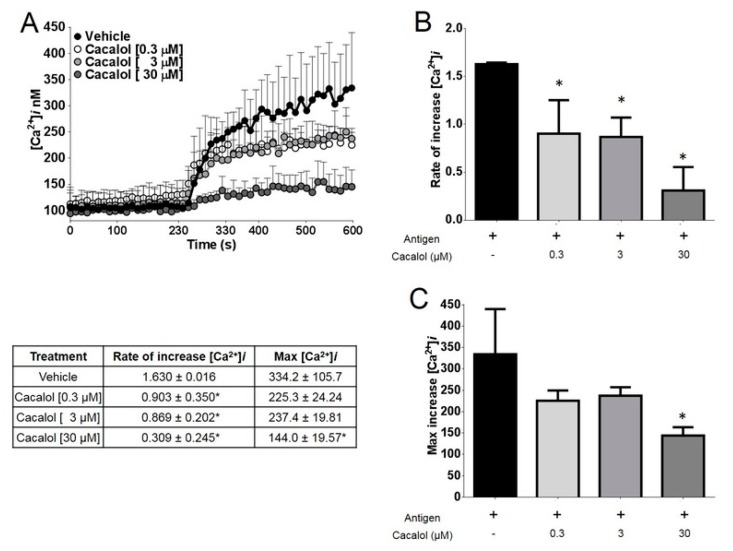
Cacalol inhibits intracellular calcium influx stimulated by FcεRI receptor in bone marrow-derived mast cells. (**A**) Representative trace of intracellular calcium rise in cells pre-treated with either vehicle or different concentrations of cacalol; (**B**) Rate of increase of [Ca^2+^]*i*; (**C**) Maximum concentration of [Ca^2+^]*i*. *n* = 3. * *p* ≤ 0.05.

Cacalol caused a 40% inhibition of the intracellular calcium [Ca^2+^]*i* mobilization required for IgE/antigen-induced degranulation at concentrations between 0.3 and 3 µM, while at 30 µM it almost completely inhibited [Ca^2+^]*i* mobilization in bone marrow-derived mast cells (Figure 3).

#### 2.2.3. Cacalol Interferes with ROS Production Induced by IgE/Ag Complexes 

**Figure 4:**
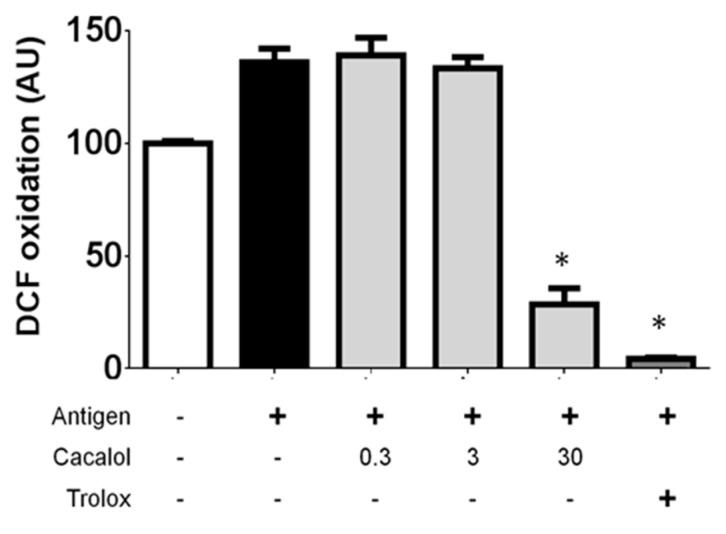
Cacalol inhibits FcεRI-triggered degranulation in BMMCs as the antioxidant Trolox does. * *p* ≤ 0.05.

Cacalol at concentrations between 30 and 300 µM almost completely inhibited the production of ROS in bone marrow-derived mast cells challenged with IgE/Ag complexes, compared to the reference antioxidant Trolox (5 mM) (Figure 4).

## 3. Discussion

Natural products have proven to be a rich source in the search and development of new drugs, either used directly or as inspiring molecules in drug development. Many of them are already in use for treatment of several diseases all over the world [16]. 

In the present study, we have used computational tools to perform a preliminary examination of the main pharmacokinetic, medicinal chemistry and toxicoinformatic properties of selected cacalolides in order to further assess their therapeutic potential. The use of computer-aided drug discovery tools has been shown to save time and resources, and to facilitate the drug discovery process [17]. First at all, none of the four compounds examined in this study yielded any toxicity parameter derived from the chemoinformatic analysis. There are no toxicological studies reported previously for these cacalolides, except for a recent in vitro study demonstrating that cacalol from *Cacalia delphiniifolia* (an Asian herbal plant) failed to induce apoptosis in human mammary epithelial cells. In contrast, this cacalolide induced apoptosis in breast cancer cells and displayed cytotoxic effects in this cellular model [18].

Our analysis also revealed that the compounds directly interacted with signaling pathways associated with the immune system including the FcεRI receptor pathway, the VEGF receptor (VEGFR2), and intracellular targets such as the activation of the PI3K-Akt kinases of the MAPKs kinases and the activation of other non-specific protein kinases.

In vitro results on FcεRI dependend-degranulation of mast cells indicate that cacalol had a higher activity than other similar sesquiterpene compounds tested. This is probably due to its unstable chemical structure, which may allow for quick decomposition into other more stable compounds by assimilating electrons derived from ROS [19] produced by FcεRI activation. The activation of membrane calcium channels linked to the Stored Operated Calcium Entry (SOCE) in mast cells may need the activation of Fc-receptors in multiple cell types of the immune system [20,21]. Interestingly, cacalol acetate, a more stable derivative generated by synthetic chemical acetylation of cacalol, is showed less inhibitory activity in the mast cells degranulation assay. In the same way, maturin acetate, a naturally occurring compound of “matarique” (*P. peltatum*), also stable under our experimental conditions, displayed an activity similar to that of cacalol acetate. This low activity was probably due to the fact that the acetyl group in both compounds conferred more stability to these molecules, which prevents them from accepting unpaired electrons from ROS [14,22]. Finally, although cacalone has been described as a more potent anti-oxidant and anti-inflammatory compound than even cacalol in the in vivo model of skin inflammation induced by TPA [12], this compound displayed poor anti-inflammatory activity in mast cells. These contrasting results are probably explained by the fact that the two models evaluated the inflammation produced by different immune cells and that cacalone was a 1:1 mixture with its putatively inactive epicacalone isomer [23]. Nevertheless, cacalol is not the only sesquiterpene possessing good inhibitory activity in the mast cell degranulation assay triggered by the FcεRI receptor. Other sesquiterpenes reported in the literature such as parthenolide, also inhibits the degranulation of mast cells by reducing the activation of NFκB and Fyn-dependent microtubule formation [24]. Additional sesquiterpenes possessing inhibitory activity similar to cacalol include deacetyleupaserrin, (3*S*,6*R*,7*R*,8*R*)-3-hydroxy-8-acetoxy-sarracenyloxygermacra-1(10),4,11(13)-trien-6,12-olide, (3*S*,6*R*,7*R*,8*R*)-3-hydroxy-8-(2′-methyl-butyroyloxy)-14-oxomelampa-1(10), 4-dien-6,12-olide and eupaformosanin, are mechanistically associated to the inhibition of p38 and Akt phosphorylation as well as to the partial inhibition of intracellular calcium mobilization ([Ca^2+^]*i*) [25]. 

## 4. Materials and Methods 

### 4.1. Reagents

All organic solvents were purchased from J.T. Baker (Radnor, PA, USA). 2′7′-dichlorofluorescein diacetate (DCF-DA), dinitrophenyl-human seric albumin (DNP-HSA), igepal, and indomethacin were obtained from Sigma-Aldrich (St. Louis, MO, USA). The antioxidant Trolox was from Calbiochem (San Diego, CA, USA), FURA-2AM from Thermo Fisher Scientific (Waltham, MA, USA). Finally, cacalol, cacalone and maturin acetate was purified as described below.

### 4.2. Isolation and Purification of Cacalolides

Cacalol and cacalone were isolated and purified from *P. decompositum* roots as previously described [26]. Cacalol acetate: acetylation of cacalol to obtain cacalol acetate was carried out as previously reported [27]. Maturin acetate was purified as described [13]. The compounds’ identities were corroborated by their ^1^H-NMR or ^13^C-NMR spectral data (see the Supplementary) obtained on a Bruker Avance III 300 MHz (Cambridge Isotope, Tewksbury, MA, USA), or High Performance Liquid Chromatography (HPLC) performed an Agilent 1200 Series Binary SL (Agilent; Santa Clara, CA, USA) using a Eclipse Plus C18, 2.1 × 100 mm, 3.5µm (Agilent; Santa Clara, CA, USA), Detector: Waters 2996 UV-Vis Photo Diode Array (Waters, Mildford, MA, USA) or Gas Chromatography coupled to Mass Spectrometry (GC-MS) was performed with a JEOL GCmate (Peabody, MA, USA) were compared with those reported in the literature, establishing that four compounds were in fact cacalol, cacalol acetate, cacalone and maturin acetate.

### 4.3. Chemical Characterization 

*Cacalol*: ^1^H-NMR (300 MHz, CHCl_3_, δ ppm) 7.24–7.22 (m, 1H), 4.78 (s, 1H), 3.24–3.01 (m, 1H), 2.98 (dd, *J* = 17.3, 4.9 Hz, 1H), 2.60 (m, 1H), 2.53 (s, 3H), 2.38 (d, 3H), 1.95–1.72 (m, 4H), 1.20–1.19 (d, *J* = 7.0 Hz, 3H); GC-MS shown peak with purity 93% at 23.9 min, this peak corresponded according to MS with the molecular weight of cacalol EI + MS *m*/*z* 230 (M^+^, 58), 215 (100) coinciding with what was previously reported [28]. 

*Cacalol acetate*: ^1^H-NMR (300 MHz, CHCl_3_, δ ppm) 7.24–7.23 (d, 1H), 3.29–3.22 (m, 1H), 2.87–2.78 (m, 1H), 2.57 (s, 3H), 2.39 (s, 3H), 2.38–2.37 (d, 3H), 1.94–1.76 (m, 4H), 1.21–1.18 (d, 3H); and ^13^C-NMR (75 MHz, (CHCl_3_, δ ppm) 168.65, 145.18, 141.42, 135.43, 131.42, 127.09, 126.86, 124.93, 116.74, 29.97, 28.92, 23.43, 21.38, 20.49, 16.59, 14.26, 11.27.

*Cacalone-epicacalone* mixture (CEM): ^1^H-NMR (300 MHz, CHCl_3_) 7.25 (d, 1H), 3.10–3.06 (m, 1H), 2.87–2.84 (m, OH), 2.49–2.44 (m, 1H), 2.19–2.18 (d, 3H), 1.73–167 (m, 5H), 1.58 (s, 3H), 1.29–1.27 (d, 3H); GC-MS shown two peak with purity 78.8% at 26.3 and 27 min, these peaks corresponded according to MS to the molecular weight of cacalone and epi-cacalone isomer. EI + MS *m*/*z* 246 (M^+^, 45 and 60), 231 (70 and 90), 204 (20 and 40), 191 (100) coinciding with what was previously reported [23,29]. The residual peaks may represent cacalol and radulifolin B based on their MS spectra. 

*Maturin acetate*: ^1^H-NMR (300 MHz, CHCl_3_, δ ppm) 11.01 (s, 1H), 8.32–8.29 (dd, 1H), 7.84 (s, 1H), 7.43–7.41 (d, 2H), 5.34 (s, 2H), 4.45 (s, 3H), 2.80 (s, 3H), 2.09 (s, 3H). HPLC shown one peak with purity 99% at 29.2 min, according to MS this peak corresponded to the molecular weight of maturin acetate. EI + MS *m*/*z* 312 (M^+^, 100), 254 (90) coinciding with what was previously reported [13]. 

### 4.4. Chemoinformatic Analysis

To calculate the bioinformatics properties of the compounds we used Osiris DataWarrior (DataWarrior V4.7.2, Idorsia Pharmaceuticals Ltd., Allschwil, Switzerland), it is a freeware software that calculates lipophilicity, expressed as compound log P (clogP), solubility in water, expressed as logS, molecular weight, drug-likeness indices and drug score; moreover, toxicological properties of the compounds may also be shown. Osiris was used to assess the possible toxicity risks as well as the aforementioned biophysical properties of cacalol, cacalol acetate, cacalone, and maturin acetate. The description of a computational medicinal chemistry can be found in a previous report [30]. 

### 4.5. Properties Calculated Using Molinspiration and PROTOX

CLogP (octanol/water partition coefficient) is calculated by the methodology developed by Molinspiration as the sum of fragment-based contributions and correction factors. Molecular polar surface area (PSA), topological polar surface area (TPSA) were calculated based in the sum of fragmented contributions. Molecular PSA has been shown to be a very good descriptor characterizing drug absorption, including intestinal absorption, bioavailability, CaCo-2 permeability, blood-brain barrier penetration, while rodent oral toxicity can be estimated by using the PROTOX web server. Based on toxic classes defined according to the globally harmonized system of classification for the labeling of chemicals six classes exist: Class I is fatal if swallowed (LD_50_ < 5 mg/kg), Class II is fatal if swallowed (LD_50_ < 50 mg/Kg), Class III toxic if swallowed (LD_50_ < 300 mg/Kg), Class IV harmful if swallowed (LD_50_ < 2000 mg/Kg), Class V may be harmful if swallowed (LD_50_ < 5000 mg/Kg), Class VI non-toxic (LD_50_ > 5000 mg/Kg). The PROTOX web server was used to perform an in silico prediction of rodent oral toxicity according to The European Parliament and Council of the European Union Regulation (REACH) [31].

### 4.6. Properties Calculated Using Swiss ADME

Swiss Bioinformatic Institute possesses a web server that calculates several ADME properties that could help to delve in the pharmaceutical properties of cacalol, cacalol acetate, cacalone, and maturin acetate in order to determine its potential to reach clinics. The complete description of the computational medicinal chemistry algorithm was published [32]. We use consensus Log P from 5 different predictions (iLOGP, XLOGP3, WLOGP, MLOGP, Silicos-IT); Log S (Silicos-IT) which is fragmental method calculated; Ghose improvement for the Lipinski rule of Five [33], and synthetic accessibility from 1 very easy to 10 very difficult, implemented in the software. 

### 4.7. Network Pharmacology

Since cacalol was the most interesting compound we wanted to identify the most important biological targets and pathways that could interact with such compound through the use of a network pharmacology approach. Predictive models were used including DRAR-CPI [34] and SEA [15]. Enrichment Analysis for Targets: Analysis of network interaction related to the first 50 targets that interact with each compound was performed using Comparative Toxicogenomics Database (CTD) [35]. The main molecular function as well as the main KEGG and Reactome pathways along with related diseases was determined. The most significantly enriched terms (*p* < 0.05, *p*-values was corrected using the Benjamini-Hochberg procedure) were listed in Table 2. 

### 4.8. Animals

C57BL/6J male mice, 25–30 g of body weight, stock number 000664, were purchased from The Jackson Laboratory (Bar Harbor, ME, USA). Mice were kept in sterile conditions, under a 12 h dark/light cycle, with free access to water and food at the Unit for the Production of Experimental Animals Laboratory (UPEAL) from Center for Research and Advanced Studies of the National Polytechnic Institute (Cinvestav) at Mexico City. All animal procedures were approved by the Cinvestav Institutional Ethics Committee (protocol number: 074-13).

### 4.9. Bone Marrow-Derived Mast Cells Isolation and Culture

Bone marrow-derived mast cells (BMMCs) were isolated from C57BL6/J mice, as described [36]. Briefly, bone marrow was extracted from the tibias and femurs of mice and cultured in RPMI 1640 (Sigma-Aldrich, St. Louis, MO, USA) medium supplemented with IL-3 (10 ng/mL; PeproTech, Rocky Hill, NJ, USA) and 10% FBS, 100 UI/mL penicillin, 100 mg/mL streptomycin, 50 mM 2-ME, and 13 nonessential amino acids (Invitrogen, Carlsbad, CA, USA). Cultures were maintained for 4 weeks, and media were changed every 5–7 days. Subsequently, BMMCs differentiation was assessed by detecting the expression of the FcεRI receptor in the plasma membrane (by flow cytometry) and by evaluating the release of β-hexosaminidase after IgE/Ag stimulation [37]. Only cultures that were 98% positive for FcεRI receptor expression and showing a dose response of β-hexosaminidase release after IgE/Ag addition were used in the study. For each experiment, BMMCs were sensitized with 100 ng/mL a monoclonal anti-DNP IgE (clone SPE-7; (Sigma-Aldrich, St. Louis, MO, USA) for 24 h at 37 °C, because this condition was shown to increase BMMCs’ responsiveness to antigen. Routinely, after sensitization, cells were collected and resuspended in fresh culture medium or Tyrode’s BSA buffer (135 mM NaCl, 5 mM KCl, 1 mM MgCl_2_, 1.8 mM CaCl_2_, 5.6 mM glucose, 0.5 g/L BSA, and 20 mM HEPES pH 7.4 for calcium experiments. For the ROS production experiments, sensitized cells were washed and resuspended in Tyrode’s Buffer without BSA.

### 4.10. Solubilization of Cacalol, Cacalol Acetate, Cacalone and Maturin Acetate for Experiments

For in vitro testing of the effects of cacalol, cacalol acetate, cacalone and maturine acetate on mast cells, the compound was dissolved in 0.1% DMSO which was also used as vehicle in all experiments.

### 4.11. β-Hexosaminidase Degranulation Assay

Two million IgE-sensitized cells were centrifuged at 500× *g* during 5 min and suspended in 1 mL Tyrode’s/BSA buffer of the following composition: 20 mM HEPES pH 7.4, 135 mM NaCl, 5 mM KCl, 1.8 mM CaCl_2_, 1 mM MgCl_2_, 5.6 mM glucose and 0.05% bovine serum albumin. Separate groups of cells were treated with vehicle, 3, 30 or 300 µM of cacalolides for 15 min and then stimulated with antigen (27 ng/mL DNP-HSA) during 30 min at 37 °C. After this treatment, cells were placed on ice for 2 min and centrifuged at 12,000× *g* for 10 min at 4 °C. Sixty µL of supernatant or (20 µL of Triton-treated cell pellet) were placed in an ELISA plate containing 40 µL of 1mM *p*-nitrophenyl-*N*-acetyl-β-d-glucosaminide (*p*-NAG), and incubated for one hour at 37 °C before the addition of 120 µL of “stop” solution (Na_2_CO_3_ 0.1M/Na_2_HCO_3_ 0.1M). β-hexosaminidase release was quantified by spectrophotometry in an ELISA plate reader (Tecan Sunrise, Männedorf, Switzerland) at 405 nm, as described [38]. 

### 4.12. Calcium Mobilization Intracellular Determination ([Ca^2+^]i)

Intracellular calcium concentrations [Ca^2+^]*i* were measured in IgE-sensitized BMMCs. Briefly, cells were collected and suspended in Tyrode’s/BSA buffer with 5 µM Fura 2-AM for 30 min at 37 °C to load the cells. Ten million of fura 2-AM-loaded BMMCs were suspended in 2 mL Tyrode’s/BSA buffer and placed in the cuvette. We monitored the fluorescence in intervals of 1.16 s until we reached 400 s on a spectrofluorometer (Fluoromax 3 Jobin Yvon, Horiba, Fukuoka, Japan) with a wavelength of 340 nm and 380 nm for excitation and 510 nm for emission respectively. Calcium concentration was calculated using the equation [Ca^2+^]*i* = Kd [(F − F_min_)/(F_max_ − F)], where Kd is the dissociation constant of calcium-Fura2AM (wavelength), F_max_ is fluorescence maximum concentration obtained by lysis of all cells with Triton [concentration] and F_min_ is the minimum fluorescence obtained by the addition of EDTA [concentration]. F was the current fluorescence of the sample. Basal fluorescence was recorded during 100 s prior to challenge with DNP-HSA antigen (27 ng/mL). [Ca^2+^]*i* was calculated with the parameters and equation described by Grynkiewicz [39]. In some experiments, we used BMMCs suspended in Ca^2+^-free Tyrode’s/BSA buffer and then added 1.8 mM CaCl_2_ (final concentration) at 200 s.

### 4.13. ROS Production and Antioxidant Activity

The cells were sensitized with IgE for 24 h prior to challenge. 2 × 10^6^ BMMC/mL were used in 1.5 mL flat top Eppendorff tubes for each treatment. They were preincubated 15 min prior to stimulating the cells with cacalolides. Trolox was used as a positive control of the antioxidant activity [40,41]. Antigen challenge (27 ng/mL of DNP-HSA) was performed for 15 min. DCF-DA [10 μM] was added for 15 min before terminating each of the stimuli, then the tubes were centrifuged at 4 °C for 5 min, the supernatant was removed and 300 µL of igepal 0.1% at 37 °C, was added by vigorous pipetting to break the cell button. The tubes were then centrifuged for 5 min at 4 °C and 200 μL were placed in a 96-well plate and read at a wavelength of 488 nm and 565 nm for excitation and emission in a model FLx800 luminometer (Biotek, Winooski, VT, USA) for 1 h, taking readings every 15 min [42]. 

### 4.14. Statistical Analysis

All data were represented as percentage mean +/− standard error of the mean using the GraphPad Prism^®^ software version 6 (GraphPad Software; La Jolla, CA, USA). Statistical analysis was performed through one way analysis of variance (ANOVA) followed by Dunnett test to compare several groups with a control. Additionally analysis with Student’s t-test and Kolmogorov-Smirnoff’s test were performed. Values of *p* ≤ 0.05 were considered statistically significant.

## 5. Conclusions

In this work we confirmed that bioinformatics tools are useful to corroborate the validity of ethnobotanical information and also to improve decision making in the identification of compounds derived from natural products with pharmacological and pharmaceutical potential. Cacalol, a molecule possessing very specific medicinal pharmacological properties and superior to those of other similar sesquiterpenes still requires more research to ensure that it remains stable under the usual environmental conditions.

## Figures and Tables

**Table 1:** Estimated physicochemical, pharmacokinetic, medicinal chemistry and toxicoinformatic properties of cacalolides *.

		Cacalol	Cacalone	Cacalol Acetate	Maturin Acetate
**Physicochemical Properties**	Log P	3.65	2.56	3.42	3.20
	Log S	−5.56	−4.04	−4.34	−5.09
	TPSA	33.37 A	50.44 A	39.44 A	65.74 A
	MW	230.30 g/mol	246.30 g/mol	272.34 g/mol	312.34 g/mol
	RB	0	0	2	5
	BD	1	1	0	0
	BA	2	3	3	5
	Molar refractivity	70.64	68.83	78.26	86.20
**Pharmacokinetic Properties**	GI absorption	High	High	High	High
	BBB permeable	Yes	Yes	Yes	Yes
	P-gp substrate	Yes	Yes	No	No
	CYP1A2 inhibitor	Yes	No	No	Yes
	CYP2C19 inhibitor	Yes	Yes	Yes	Yes
	CYP2C9 inhibitor	No	No	No	Yes
	CYP2D6 Inhibitor	No	No	No	No
	CYP3A4 inhibitor	No	No	No	Yes
	Log Kp (Skin permeation)	−4.62 cm/s	−6.14 cm/s	−5.79 cm/s	−5.99 cm/s
**Medicinal Chemistry Properties**	Lipinski	Yes	Yes	Yes	Yes
	Ghose	Yes	Yes	Yes	Yes
	Bioavailability Score	0.55	0.55	0.55	0.55
	Lead-likeness	No, 2 Violations	No, 1 violation	Yes	Yes
	Synthetic accessibility	2.75	4.32	4.72	3.77
**Toxicoinformatic Properties**	Toxicity Class	5	4	5	5
	Mutagenic	None	None	None	None
	Tumorigenic	None	None	None	None
	Irritant	None	None	High	None
	Reproductive effects	None	None	None	None
	Possible Toxic Target	None	None	None	Amine oxidase prostaglandin G/H synthase 1
	Toxic Fragments	None	None	None	None

* Theoretical values. Abbreviations: GI—Gastrointestinal absorption; BBB—Blood brain barrier; P—gp-Glycoprotein P; GPCR—Receptor of protein G; MW—Molecular weight; RB—Rotatable bonds; BD—Number of H donors; BA—Number of H-bond acceptors; TPSA—Total polar surface area.

**Table 2:** Enrichment analysis (GO and Pathway) of the main 50 targets that interact with cacalol using network pharmacology approaches *.

Compound	Pathway or GO Term	Count	*p*-Value	Data Base
Cacalol	Innate immune system (hsa-168249)	24	2.43 × 10^−20^	Reactome
	VEGFA-VEGFR2 pathway (hsa-4420097)	12	4.42 × 10^−12^	Reactome
	Fc epsilon receptor signaling (hsa-2454202)	12	4.42 × 10^−12^	Reactome
	PI3K-Akt pathway (hsa04151)	12	4.91 × 10^−12^	KEGG ^1^
	MAPK signaling pathway (hsa04010)	11	7.89 × 10^−12^	KEGG ^1^
	Protein kinase activity (GO:0004672)	26	1.58 × 10^−31^	GO-MF ^2^

* Theoretical values. ^1^ Kyoto Encyclopedia of genes and genomes. ^2^ Gene Ontology-Molecular Function.

## Supplementary Materials

Nuclear Magnetic Resonance (NMR) and Gas Chromatography–Mass Spectrometry (GC-MS) of cacalol, cacalol acetate, cacalone and maturine acetate.
