# Supplementary material for: Chemoinformatic Analysis of Selected Cacalolides from Psacalium decompositum (A. Gray) H. Rob. & Brettell and Psacalium peltatum (Kunth) Cass. and Their Effects on FcεRI-Dependent Degranulation in Mast Cells
Source: Molecules. 2018 Dec 19;23(12):3367. doi: 10.3390/molecules23123367 (PMC6321304; doi:10.3390/molecules23123367)

Supplementary figure 1-A: 300 MHz ¹H-Nuclear Magnetic Resonance (NMR) of cacalol.

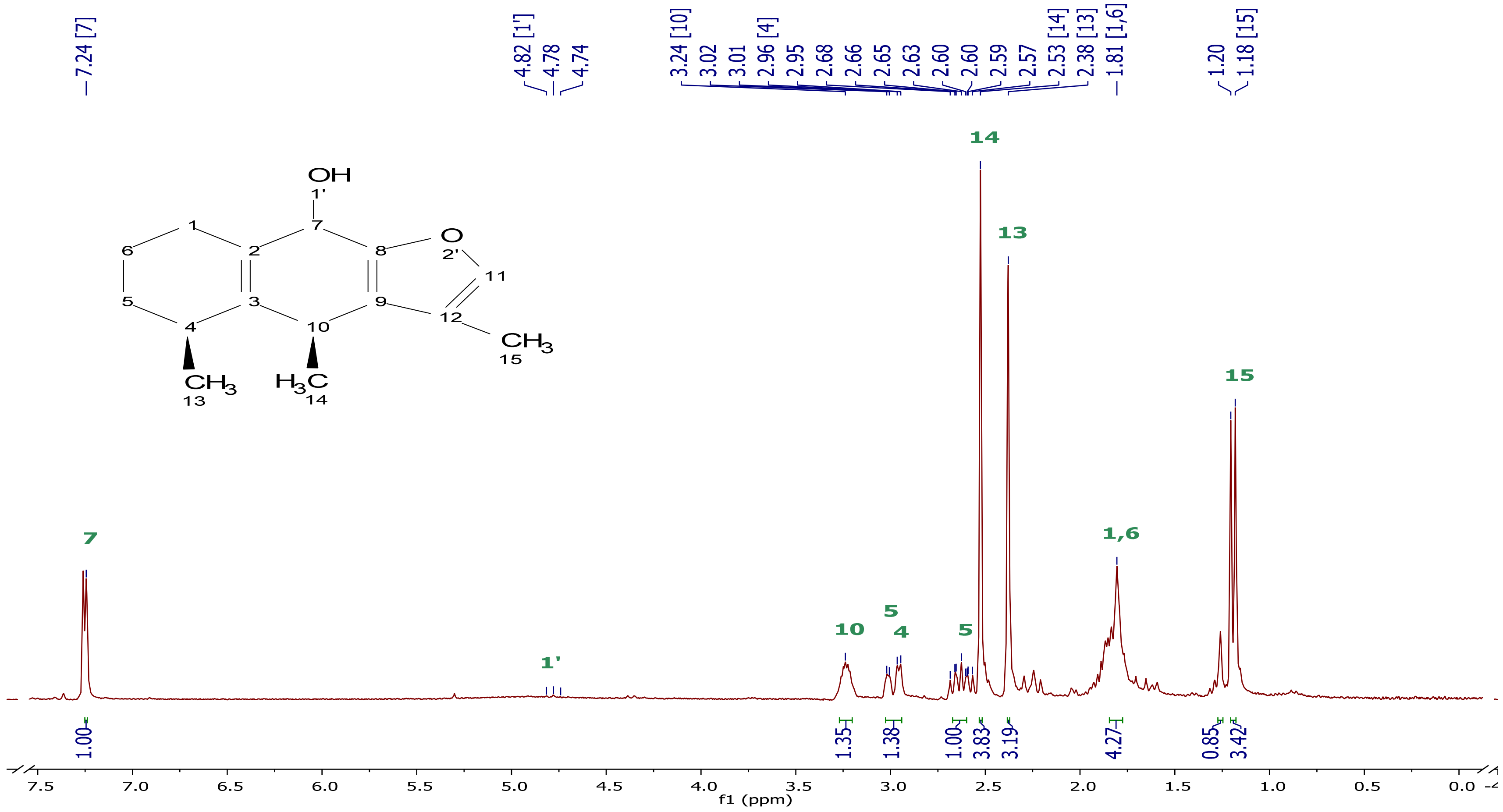

Supplementary figure 1-B. Gas Chromatography–Mass Spectrometry (GC-MS) of cacalol.

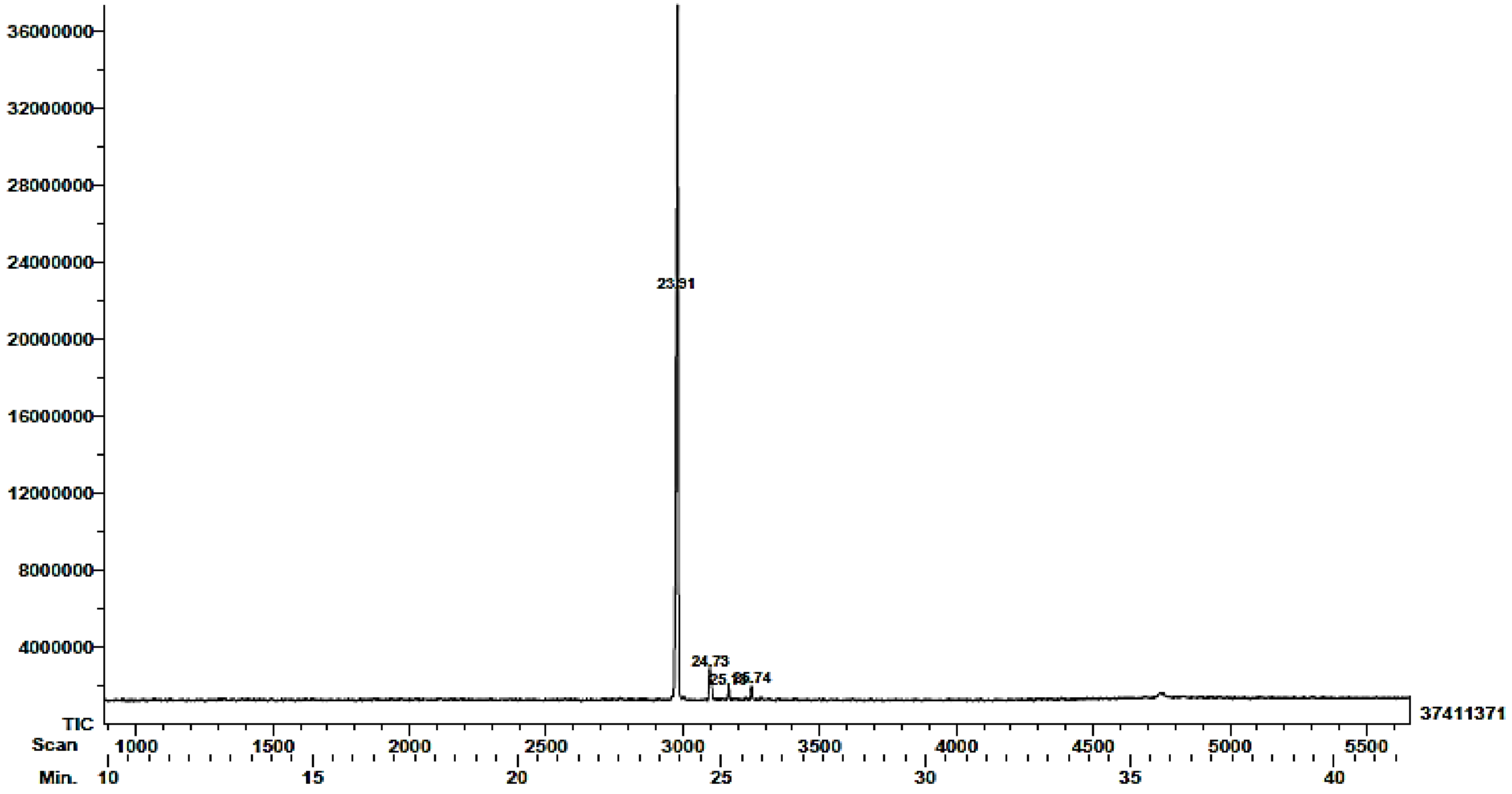

Supplementary figure 1-C. Gas Chromatography Mass Spectrometry (GC-MS) of peak 23.91 min reference to cacalol.

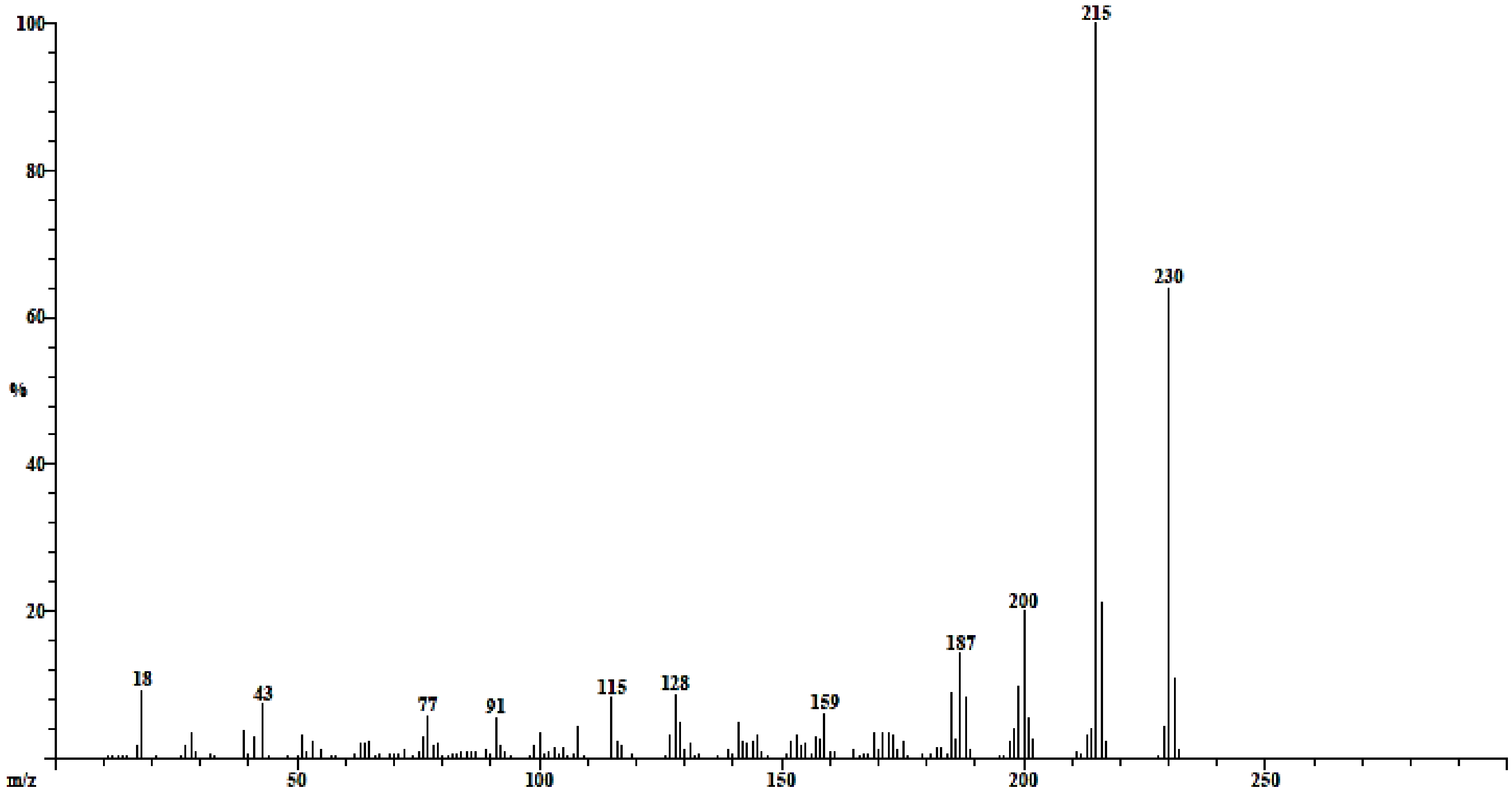

Supplementary figure 2-A: 1HNMR 300 MHz of cacalol acetate.

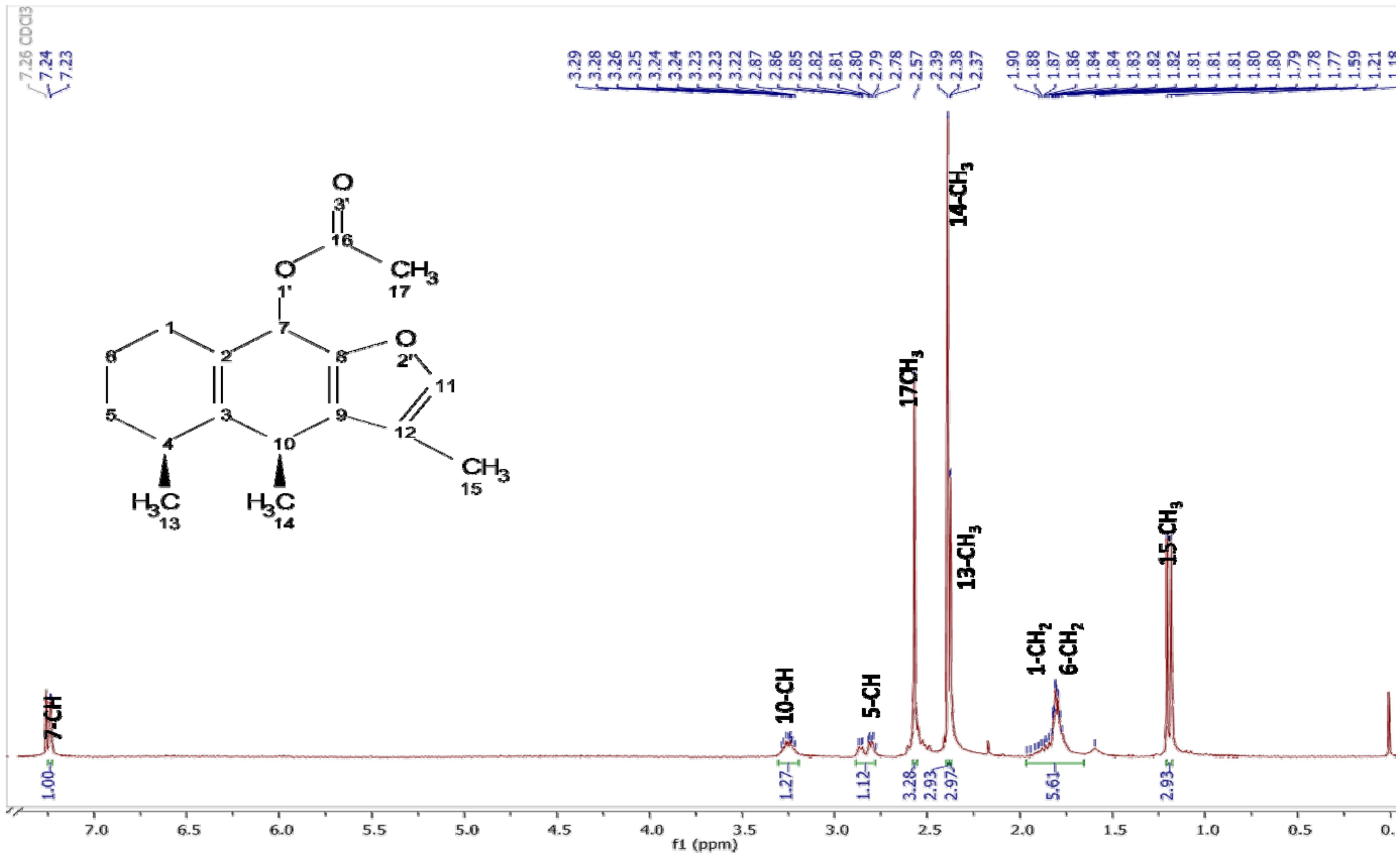

Supplementary figure 2-B: 300 MHz 13C-Nuclear Magnetic Resonance (NMR) of cacalol acetate.

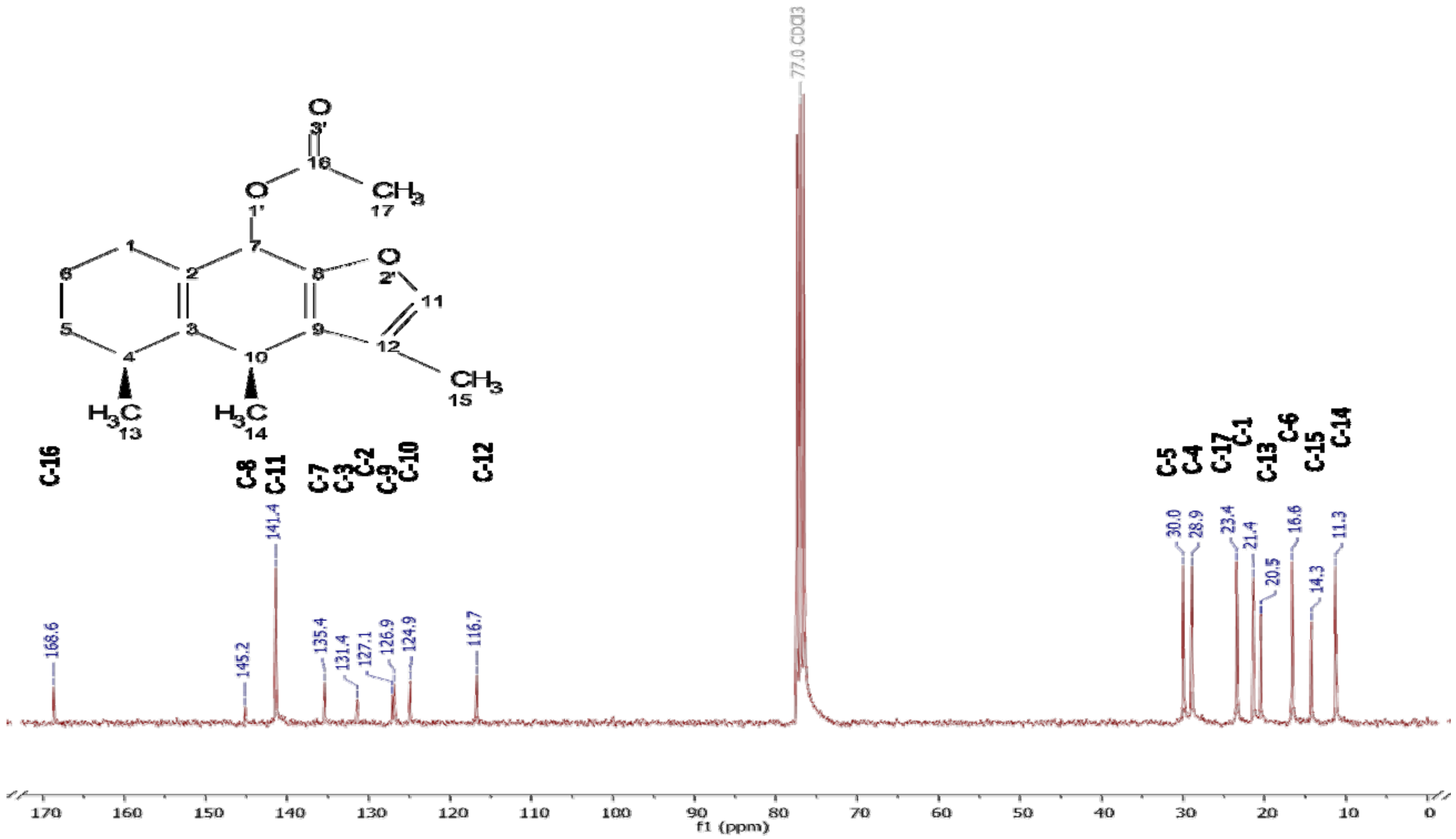

Supplementary figure 3-A: 300 MHz 1H-Nuclear Magnetic Resonance (NMR) of cacalone.

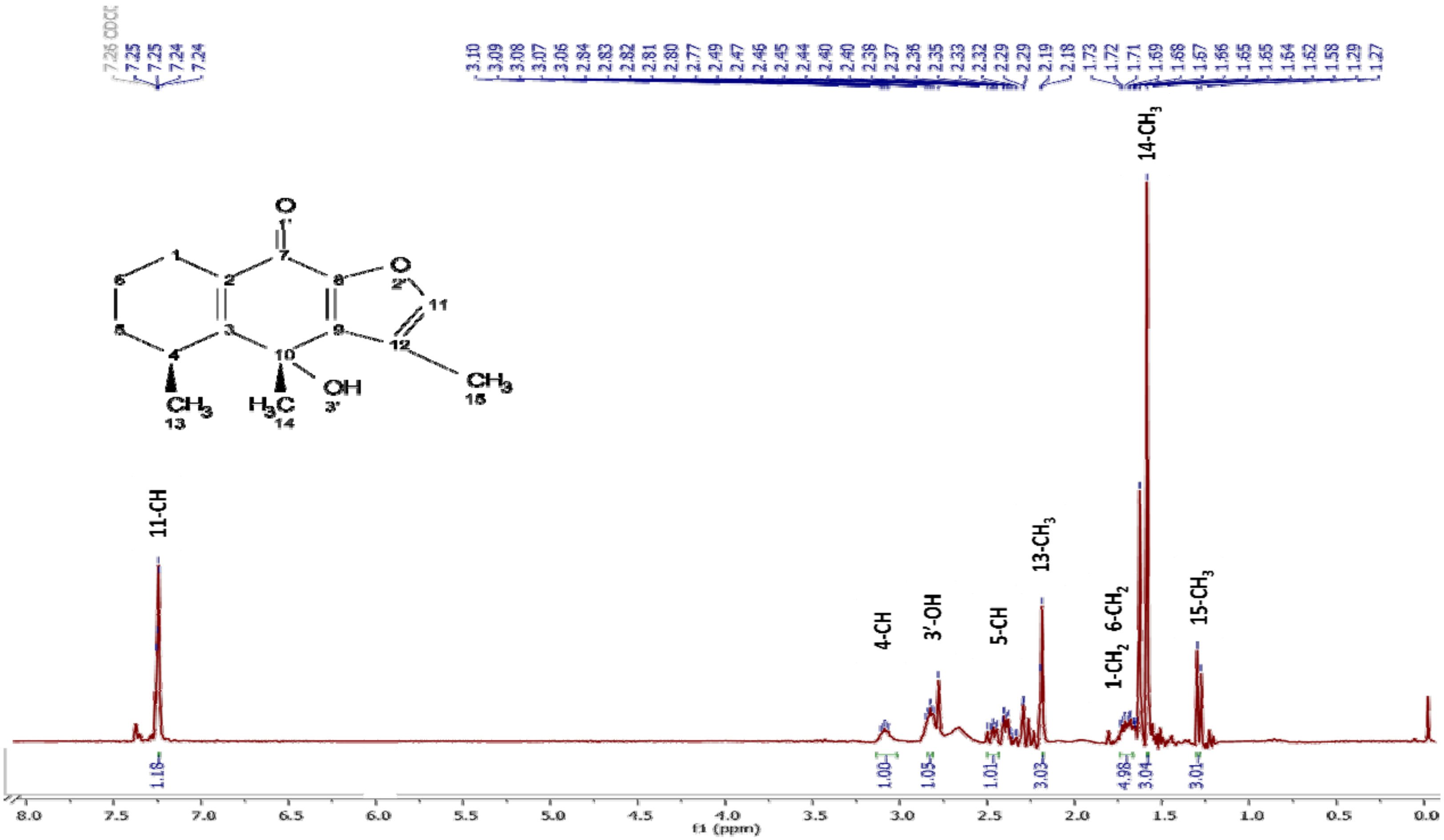

Supplementary figure 3-B: Gas Chromatography–Mass Spectrometry (GC-MS) of cacalone.

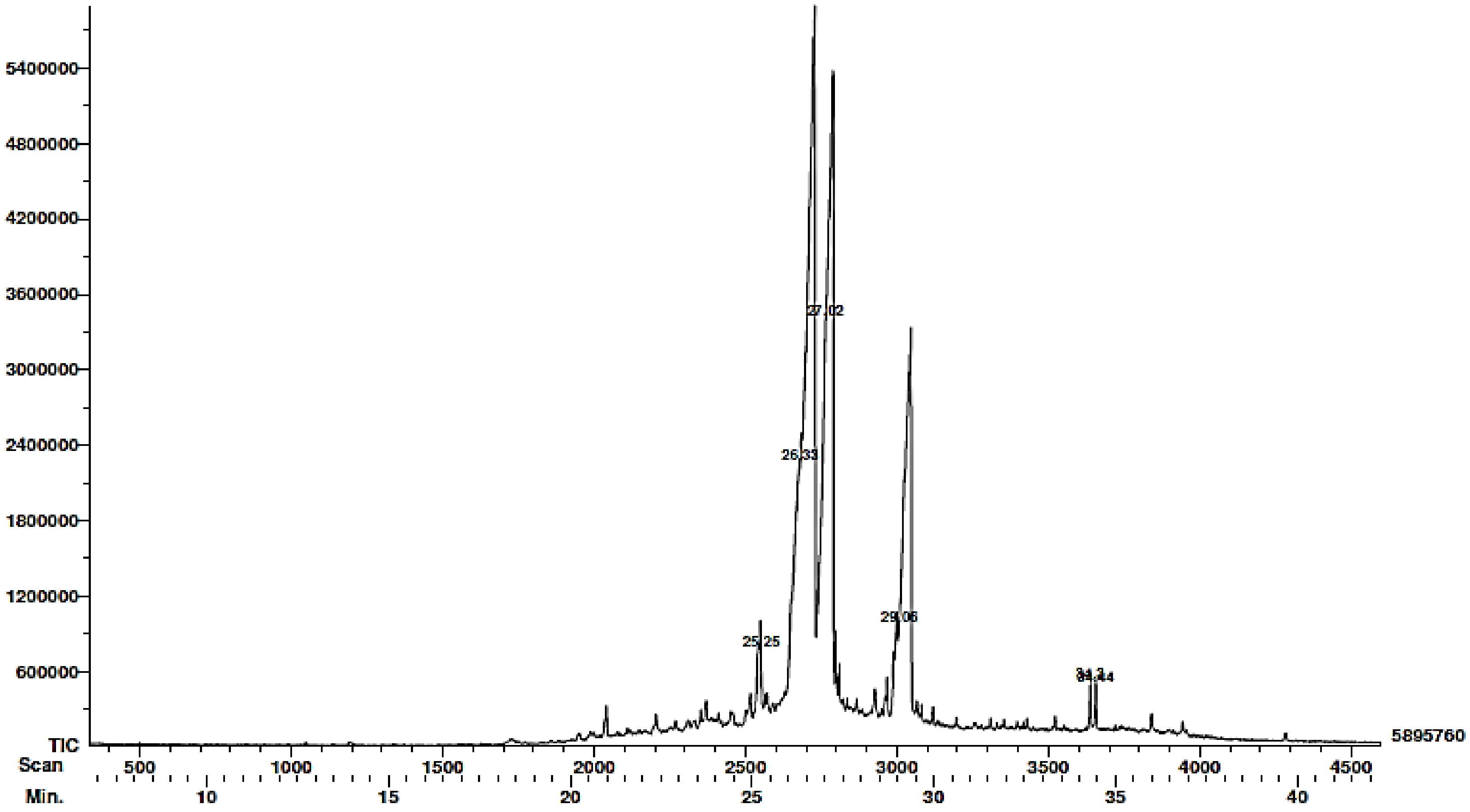

Supplementary figure 4-A: ^{1}H-NMR 300 MHz of maturin acetate.

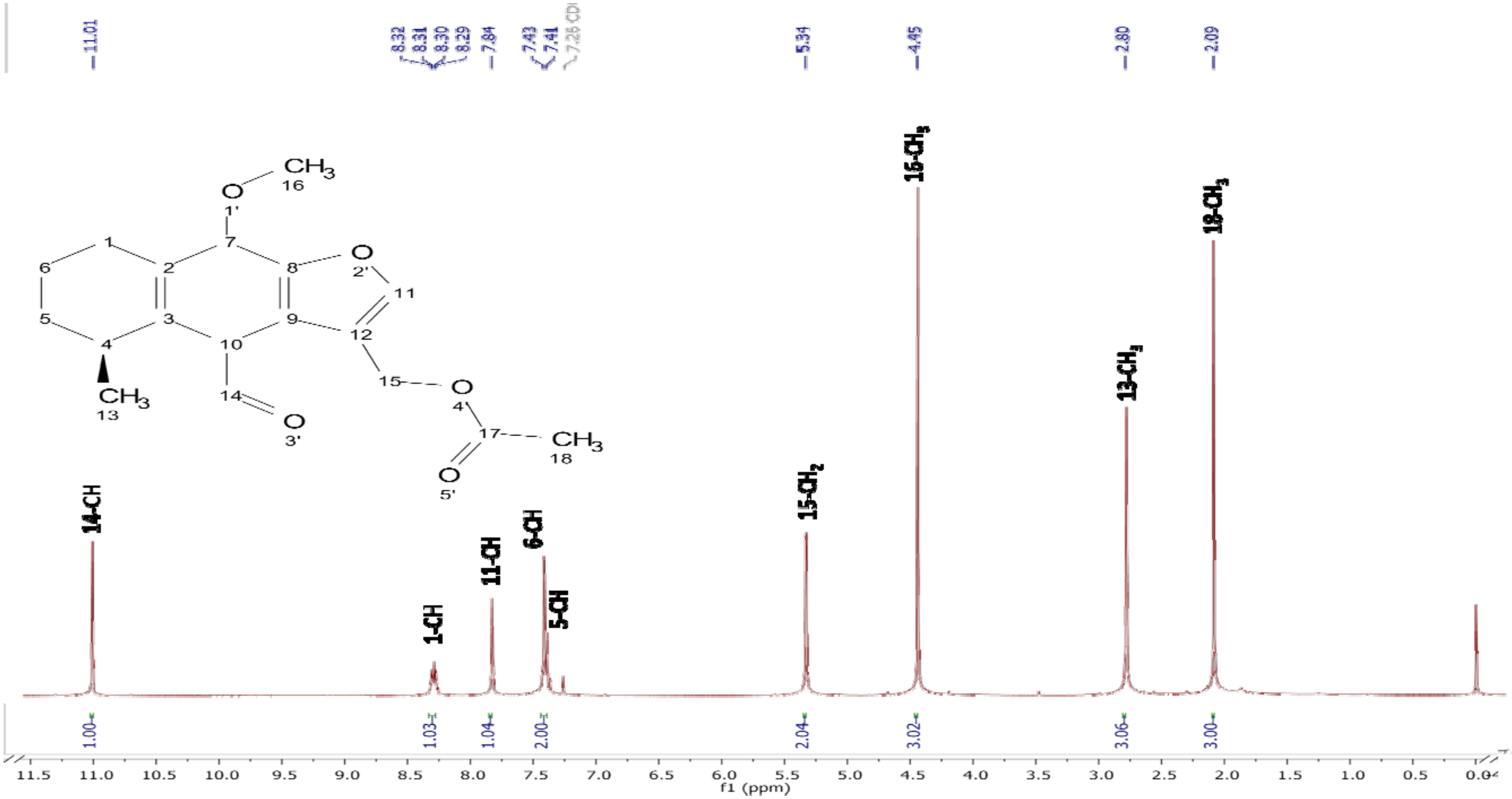

Supplementary figure 4-B: HPLC of maturin acetate.

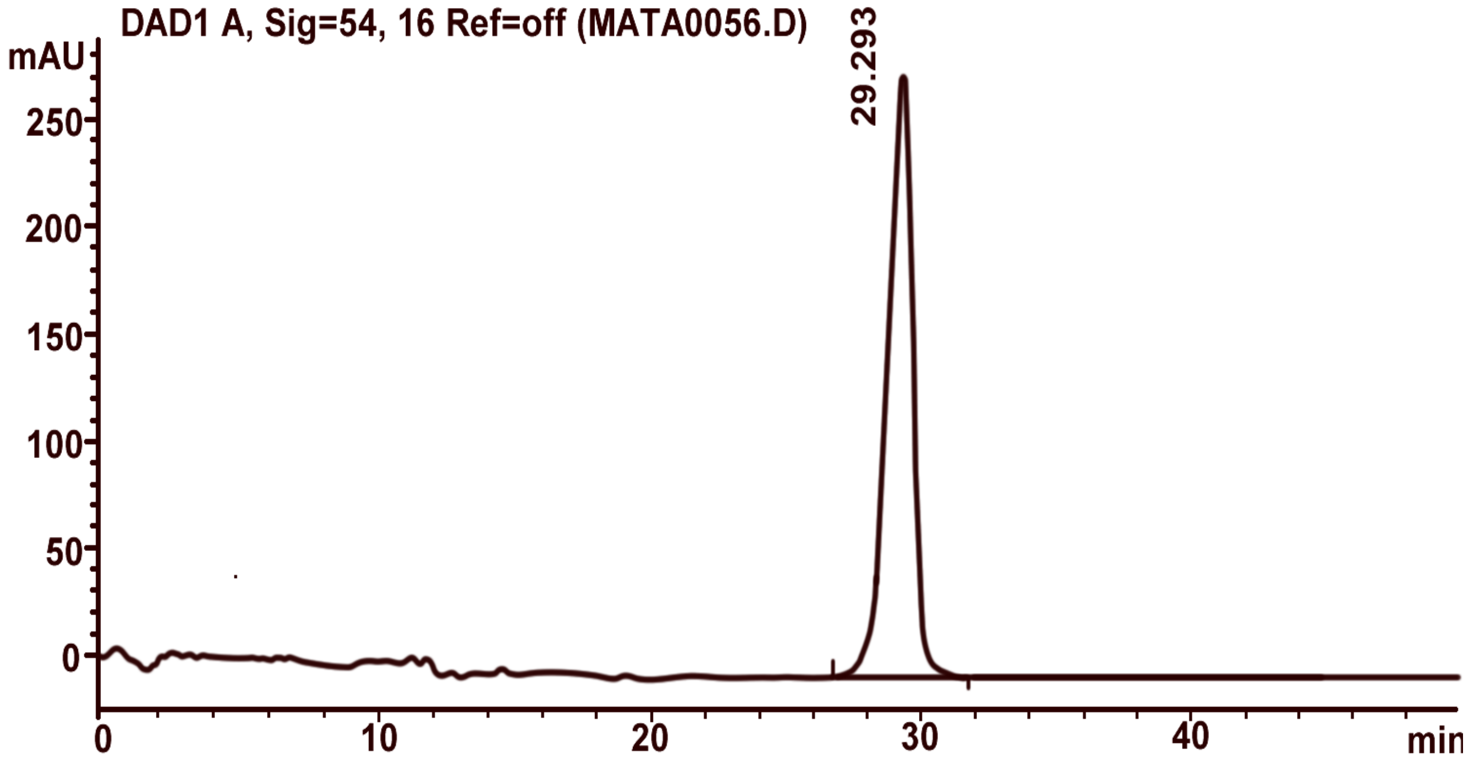

Figure 4-C: Mass Spectrometry of maturin acetate.

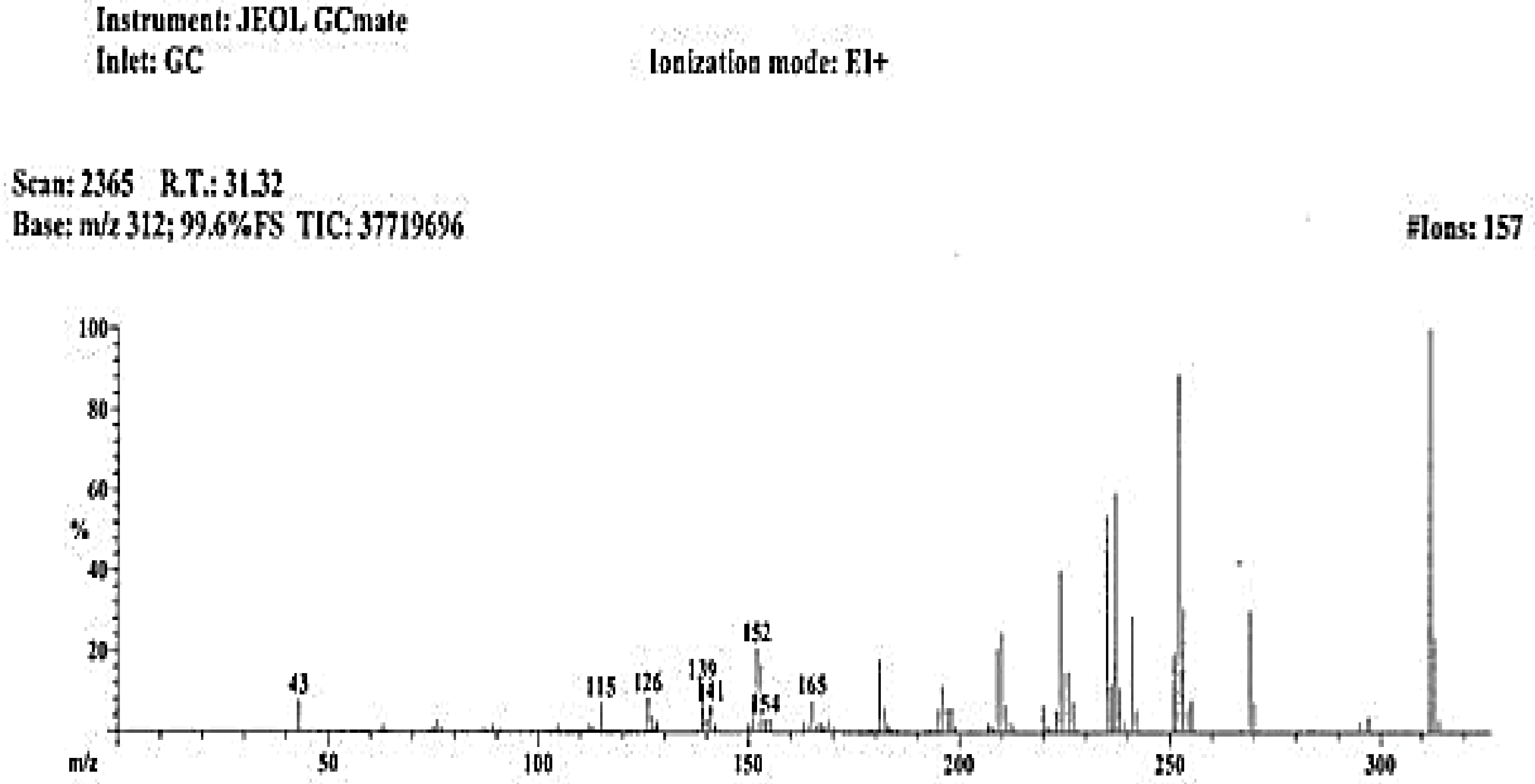

Supplement: Supplementary file 1 [file molecules-23-03367-s001.pdf]
